# The effect of perceived social support and health literacy on parental COVID-19 vaccine hesitation in preschool children: a cross-sectional study

**DOI:** 10.1038/s41598-024-53806-6

**Published:** 2024-02-08

**Authors:** Jiayue Chen, Quqing Wang, Nan Jiang, Yuxin Zhang, Ting Wang, He Cao, Yongyi Liu, Yonghui Yang, Jiwei Wang

**Affiliations:** 1 Huacao Community Health Service Center, Minhang District, Shanghai, China; 2 Key Lab of Health Technology Assessment of Ministry of Health, School of Public Health, Fudan University, 130 Dong-An Road, Shanghai, 200032 China; 3 The Joseph L. Mailman School of Public Health, Columbia University in the City of New York, 1130 Amsterdam Ave, New York, NY 10027 USA

**Keywords:** Risk factors, Disease prevention, Lifestyle modification, Preventive medicine

## Abstract

Children are generally susceptible to COVID-19, and infection with COVID-19 may cause serious harm to children. COVID-19 vaccination is an effective way to prevent infection at present, and many factors affect children's COVID-19 vaccination. This study aimed to explore the effects of perceived social support and health literacy on hesitancy towards first and second vaccine dose. This cross-sectional study was conducted in the Minhang District of Shanghai, China, in October 2022. A total of 1150 parents of preschool children from 10 kindergartens participated. The survey encompassed four sections, capturing data on sociodemographic attributes, health literacy, perceived social support, and parental COVID-19 vaccine hesitancy. Health literacy was measured using a self-designed questionnaire consisting of four dimensions. Perceived social support was assessed using the MSPSS questionnaire. Hierarchical multiple logistic regression was used to examine the relationship between the independent variables and parental hesitancy towards the first and second doses of COVID-19 vaccine. Parental hesitancy rate for the first dose of the COVID-19 vaccine was 69.6%, and for the second dose, it was 33.1%. The final integrated model showed that parental hesitancy towards the first and the second dose of COVID-19 vaccine was associated with parental educational level, allergy in children, information decision-making and information comprehension ability, perceived social support from family and friends. Health literacy and perceived social support are influence factors for parental hesitancy towards COVID-19 vaccine for preschool children. The findings will provide insights for future intervention studies on COVID-19 vaccine hesitancy and inform the development of vaccination policies.

## Introduction

At present, the COVID-19 virus continues its global dissemination, posing a pervasive susceptibility across all age cohorts. Since November 2021, Omicron strains have spread widely worldwide^1^. In 2022, Shanghai experienced two rounds of COVID-19 outbreaks, from March to June and in December. According to the Shanghai Center for Disease Control and Prevention, the local outbreak of the Omicron subline age BA.2 in Shanghai since March 2022 resulted in over 0.6 million laboratory confirmed infections in early June^2^. As of April 30, 2022, the Shanghai Municipal Health Commission has reported 12,707 positive cases among children under 6 years old. This accounts for approximately 2.4% of the total number of infections^3^. Contracting severe acute respiratory syndrome coronavirus 2 (SARS-CoV-2) can yield enduring effects on the health of children^3^. Studies reveal that the coronavirus in pediatric COVID-19 patients frequently affects multiple systems, including the respiratory system, nervous system and cardiovascular system^4,5^. Additionally, children under 5 years old are more likely to experience fatigue and loss of taste and smell for more than 4 weeks after infection^6,7^. Concurrently, instances of severe illness and mortality among children due to COVID-19 are still on the rise^8^. As of August 2023, a research analysis by the University of Hong Kong involving 1144 children aged 11 or younger hospitalized due to COVID-19 indicated that the rates of severe illness and mortality among Hong Kong children infected with COVID-19 were 1.8% and 0.2%, respectively^9,10^.

Vaccination has been proven to be effective in preventing SARS-CoV-2 infection in children^11–13^. In February 2022, the Centers for Disease Control and Prevention (CDC) approved the use of the BNT162b2 vaccine for children aged 6 months to 11 years old^13,14^. In June 2022, a clinical trial of the BNT162b2 vaccine against the omicron variant in children aged 6 months to 4 years old confirmed its safety and effectiveness^13,15^, with results showing a vaccine efficacy against COVID-19 from 7 days after vaccination of 73.2%^16^. The results of a randomized, double-blind, controlled, phase 1/2 trial of the inactivated COVID-19 vaccine BBIBP-CorV conducted in China have shown that the inactivated COVID-19 vaccine is safe and well tolerated at all tested dose levels in participants aged 3–17 years^11^. A study on vaccine efficacy, encompassing 48,243 cases of individuals infected with the BA.2 variant of the COVID-19 virus in Shanghai from March to May 2022, revealed that domestically produced COVID-19 vaccines offer limited protection. They prevent asymptomatic infections from progressing to mild-to-moderate illness and provide enduring protection against non-severe illnesses caused by the Omicron BA variant, preventing them from progressing to severe conditions^2^. ^151515^As of January 4, 2023, 23.82% of children under 3 years old in Hong Kong have received 1 dose of the COVID-19 vaccine^17^. As of April 27, 2023, 77.6% of children and adolescents under 12 years old in the United States have completed the full course of COVID-19 vaccination, and 19.1% have completed booster dose vaccination^18^. As of April 2021, three COVID-19 vaccines have been approved and widely used in the Chinese population aged 18 and above^19^. These include inactivated vaccines (Sinopharm and Sinovac), adenovirus vector vaccines (CanSinoBio), and protein subunit vaccines (Zifivax)^20^. In November 2021, the Chinese mainland approved the free vaccination of the 3–17 age group with the inactivated COVID-19 vaccine. The vaccination regimen includes two doses administered at an interval of 3–8 weeks. Parents of children adhere to the principles of informed consent and voluntary vaccination. Booster doses are only recommended for adults aged 18 years and older in Shanghai^2^.

Vaccine hesitancy(VH) is defined as "delay in acceptance or refusal of vaccines despite the availability of vaccination services"^21^. Vaccine hesitancy occurs on the continuum between high vaccine demand and complete vaccine refusal, i.e. no demand for available and offered vaccines^21^. Parental vaccine hesitancy, defined as parents’ delay in acceptance or refusal of vaccines despite their availability for their children, might constitute an important obstacle to vaccination^22^. It is a multifactorial phenomenon involving individual, group, and environmental factors^21,23,24^, and many studies have reported on vaccine hesitancy among parents regarding their children's COVID-19 vaccination^25–27^. Parental vaccine hesitancy for their children's COVID-19 vaccination has been found to be associated with parental sociodemographic characteristics^28^, the child's susceptibility to COVID-19^29^, and parental self-efficacy, among other factors^30,31^. Lack of trust in the safety and efficacy of COVID-19 vaccines is an important influencing factor for parental vaccine hesitancy^29,32^, including a lack of understanding of the benefits of vaccination and the spread of vaccine conspiracy theories on social media^33^.

Parents' knowledge and attitudes towards vaccine-related information determine their willingness to vaccinate their children against COVID-19, and increasing parents' confidence in vaccinating their children can improve vaccine hesitancy^33,34^. It has been reported that social support from family, friends, colleagues, and the community is a favorable factor for improving parental self-efficacy and confidence in vaccination^30,34^, and a study of parents vaccinating children against COVID-19 also reported this result^32^.

Health literacy is defined as "the degree to which individuals have the capacity to obtain, process, and understand basic health information and services needed to make appropriate health decisions"^25^. The basic assumption underlying this definition is that individuals with satisfactory levels of health literacy are more effective at managing their health^25^. This indicates that health literacy is associated with literacy skills, requiring individual knowledge, motivation, and the ability to access, comprehend, assess, and apply health information. This is essential for making judgments and decisions regarding healthcare, disease prevention, and health promotion in daily life^35,36^. Parental health literacy is related to their acquisition and cognition of information about COVID-19 vaccines, with higher health literacy scores being associated with more positive attitudes towards COVID-19 vaccination^33^, and less hesitancy towards receiving the vaccine^37^. However, some studies have also shown that health literacy may lead to higher risk calculation^37^. Studies have shown that improving health literacy can play an important role in individuals' choice to participate in activities related to their health^38^. The acquisition, comprehension, communication, and decision-making of information related to COVID-19 and its vaccines affect parental vaccine hesitancy towards their children^25,35,36,38^.

Perceived social support (PSS) refers to how individuals view friends, family members, and others as sources of material, psychological, and overall support^39^. Existing literature indicates that support individuals receive from their social networks (such as family and friends) can significantly impact health decisions and actions^31,34^. Moreover, perceived social support is often a fundamental element in shaping individuals' confidence and self-efficacy in making health decisions. It contributes to the formation of attitudes and beliefs regarding health behaviors^34^. Perceived social support theory has been widely used in many studies related to health behaviors^40^. There is evidence to suggest that social support can have beneficial effects on various health behaviors^34^. For example, perceived social support has been found to enhance the life satisfaction and well-being of cervical cancer patients^41^, and a study has reported that high levels of social support can facilitate HPV vaccination^42^. Providers of social support can serve as role models in promoting health behaviors, and they may support behavior change by offering instrumental assistance or emotional encouragement^31^. When making significant health choices, individuals need to acquire accurate information and engage in conversations with knowledgeable individuals in order to have a sense of control over their decisions^34^.

This study aims to assess the impact of parental perceived social support and health literacy on vaccine hesitancy for COVID-19 vaccination among preschool children. By investigating the factors contributing to vaccine hesitancy, the study intends to explore effective intervention measures for reducing vaccine hesitancy. The findings will provide insights for future intervention studies on COVID-19 vaccine hesitancy and inform the development of vaccination policies.

## Methods

### Study design and data collection

This cross-sectional study recruited parents of preschool-aged children from ten kindergartens in Huacao Town, Minhang District, Shanghai, in October 2022. Huacao Town had a total of 3396 preschool children aged 3–6 years. According to the sample size calculation for cross-sectional studies, with an estimated vaccine hesitation rate of 35%^26^ a significance level of 0.05, an allowable error of 0.035, a minimum sample size of 743 was required. Considering an inefficiency rate of 20%, at least 892 participants needed to be recruited. Cluster sampling was employed in this study, and recruitment invitations were sent to parents of the ten kindergartens in Huacao Town. The study utilized the online survey platform Questionnaire Star to create the web-based survey questionnaire. On October 30, 2022, the survey questionnaire was published through WeChat, a popular Chinese social media platform, in the WeChat groups of each kindergarten. Kindergarten teachers forwarded the questionnaire link to the parents in their respective class WeChat groups and briefly introduced the research purpose. Parents completed the questionnaire online. As of November 6, 2022, a total of 1901 questionnaires were received, with a response rate of 55.98%. To ensure the quality of the responses, two quality control questions were included. Only questionnaires with correct answers to both quality control questions were considered valid. After screening based on the quality control questions, 1720 valid questionnaires were obtained. The survey questionnaire initially inquired about the vaccination status of preschool-aged children for COVID-19. Among the respondents, 570 parents reported that their children had completed the full course of COVID-19 vaccination. A survey was conducted on the remaining 1,150 parents of preschool children, including 905 parents who had never vaccinated their preschool children against COVID-19 and 245 parents whose children had not completed the full vaccination course. It is hypothesized that these groups of parents may have vaccine hesitancy. Additionally, the questionnaire was designed to allow submission only after answering all the questions, and each account could submit the questionnaire only once. Before the commencement of the survey, all participants were provided access to an electronic version of the informed consent document. This document delineated the purpose and significance of the study, informing them that they would be contributing data for scientific research. It assured participants of the confidentiality and anonymity of all data, emphasizing that the information would solely be used for research purposes. Parents were given the autonomy to decide whether or not to participate in the survey. Upon clicking the "Agree" button, participants proceeded to engage in the survey. The study obtained the participants' consent and their responses to the questionnaire. This study was approved by the Ethics Committee of the School of Public Health, Fudan University (International Registration Number: IRB00002408 and FWA00002399). All methods were performed in accordance with the relevant guidelines and regulations.

### Measurement tools

#### Sociodemographic characteristics

Sociodemographic data were collected from each participant, including parental age, child’s kindergarten year, parental educational level, monthly household income per capita, history of allergic diseases in the child, and presence of congenital diseases in the child.

#### Health literacy

Following previous studies^36,38^, we measured parents' COVID-19 vaccine health literacy using a self-developed questionnaire. The questionnaire consisted of 8 items, assessing four dimensions: information acquisition, information comprehension, information communication, and information decision-making. The information acquisition dimension measured parents' ability to obtain information about COVID-19 vaccination for their preschool children. The information comprehension dimension assessed parents' understanding of vaccine adverse reactions and the significance of COVID-19 vaccination for preschool children. This is done using items such as, "When exposed to information about vaccinating preschool children against COVID-19, please rate the difficulty of your understanding of vaccine-related content (including the protective effect against COVID-19, side effects, and explanations of contraindications, etc.)" The information communication dimension measured the extent of information exchange between parents and healthcare professionals as well as their social network. The information decision-making dimension evaluated parents' capability to make vaccination decisions for their children. This is assessed using items such as, "Please assess the difficulty of deciding whether to have your child vaccinated against COVID-19 based on relevant information." Each item was rated on a 5-point Likert scale (1 = "very difficult" to 5 = "very easy"). Higher scores reflected better health literacy regarding COVID-19 vaccination for preschool children. In this study, the Cronbach's alpha coefficient for this questionnaire was 0.749.

### Perceived social support

We assessed parents' perceived social support (PSS) using the Multidimensional Scale of Perceived Social Support (MSPSS). The MSPSS is a self-report questionnaire developed by Zimet et al.^43^ and consists of 12 items. It measures participants' perceived social support from three informal sources: family (PSS-Fa), friends (PSS-Fr), and significant others (PSSO). Participants rated the items on a 7-point Likert scale (1 = "strongly disagree" to 7 = "strongly agree"). Example items include "I receive emotional help and support from my family." We treated the scores as continuous variables and computed the average score (ranging from 1 to 7) to assess the level of PSS. Higher scores indicate a higher perceived level of social support. In this study, the Cronbach's alpha for this scale was 0.940, indicating good internal consistency.

### Vaccine hesitancy

Refer to previous studies on vaccine hesitancy question Settings^24,26^, COVID-19 vaccine hesitancy was measured via the following questions: For parents of children who have not received any dose of the COVID-19 vaccine, they were asked, "Do you accept the first dose of COVID-19 vaccination for your child?" For parents of children who have received the first dose of the COVID-19 vaccine, they were asked, "Do you accept the second dose of COVID-19 vaccination for your child?" The response options were “1 = accept” and “2 = uncertain”. Choosing "uncertain" is defined as hesitancy, and choosing "accept" is defined as no hesitancy. Option 1 was defined as the absence of vaccine hesitancy, while Option 2 indicated the presence of vaccine hesitancy. Furthermore, for parents who selected the uncertain option, we asked them to provide the reasons for their uncertainty. This question allowed for multiple selections, including options such as "I am concerned about the vaccine's effectiveness in protecting my child," "I am worried about potential side effects of the vaccine," "There have been many negative news or reports about the vaccine," "My child is not suitable for vaccination due to poor health," "My child is healthy and does not need vaccination," and "My child is allergic to vaccines," as well as an "other" option.

### Statistical analysis

Descriptive statistics were used to analyze the sociodemographic characteristics of the study population. Qualitative variables were expressed as numbers and percentages, while quantitative variables were presented as means and standard deviations. Cronbach's alpha was computed to assess the internal consistency of the perceived social support and vaccine hesitancy questionnaires. The chi-square test was employed for analyzing the relationships between variables. Following previous research^26^ tolerance values > 0.1 and VIF values < 10 were used to check for multicollinearity. Hierarchical multivariate logistic regression was performed to analyze vaccine hesitancy for the first and second doses of COVID-19 vaccination in preschool children. Model 1 consisted of significant sociodemographic variables identified through univariate analysis. Model 2 included perceived social support variables. Model 3 incorporated health literacy variables. The inclusion order of variables in the model was based on two prior assumptions. First, perceived social support can explain additional variation beyond sociodemographic factors. Second, after accounting for individual factors and the variance explained by perceived social support, an increase in variance can be explained by health literacy variables. All statistical analyses were conducted using IBM SPSS Statistics 20 software (IBM Corp.), and a two-sided significance level was set at 0.05.

### Ethics approval and consent to participate

All participants were informed that they were contributing data to a scientific study, and all data were treated as confidential and anonymous, solely used for research purposes. Informed consent was obtained from the participants. The study obtained the participants' consent and their responses to the questionnaire. This study was approved by the Ethics Committee of the School of Public Health, Fudan University (International Registration Number: IRB00002408 and FWA00002399). All methods were performed in accordance with the relevant guidelines and regulations.

## Results

### Sociodemographic factors

Table 1 summarizes the sociodemographic characteristics of the participants. A total of 1,150 parents of preschool children were included in this study, among whom 905 preschool children had never received the first dose of the COVID-19 vaccine. Over two-thirds of parents (66.52%) were between 30 and 40 years old, and 50.06% of preschool children attended kindergarten in the lower grades. More than half of the parents (58.01%) had a college or undergraduate degree, and 40.89% of families had a monthly per capita income between 5000 and 9999. The majority of children (84.97%) did not have any allergies, and 98.90% did not have any congenital diseases.

**Table 1:** Correlation analysis between sociodemographic factors and parents' hesitation about COVID-19 vaccine in preschool children.

	First does of COVID-19 Vaccine				Second does of COVID-19 Vaccine			
	Total n = 905 n(%)	Hesitancy n = 630 n(%)	No Hesitancy n = 275 n(%)	*P*	Total n = 245 n(%)	Hesitancy n = 81 n(%)	No Hesitancy n = 164 n(%)	*P*
Parental age				0.006				< 0.001
< 30 years	248(27.40%)	153(61.69%)	95(38.31%)		77(31.43%)	14(18.18%)	63(81.82%)	
30–40 years	602(66.52%)	436(72.43%)	166(27.57%)		154(62.86%)	58(37.66%)	96(62.34%)	
> 40 years	55(6.08%)	41(74.55%)	14(25.45%)		14(5.71%)	9(64.29%)	5(35.71%)	
Kindergarten year				0.043				0.126
Primary class	453(50.06%)	316(69.76%)	137(30.24%)		13(5.31%)	1(7.69%)	12(92.31%)	
Junior class	245(22.07%)	158(64.49%)	87(35.51%)		109(44.49%)	39(35.78%)	70(64.22%)	
Senior classes	207(22.87%)	156(75.36%)	51(24.64%)		123(50.20%)	41(33.33%)	82(66.67%)	
Parental educational level				< 0.001				0.928
Junior or below	137(15.14%)	78(56.93%)	59(43.07%)		60(24.49%)	19(31.67%)	41(68.33%)	
High school	211(23.31%)	122(57.82%)	89(42.18%)		73(29.80%)	26(35.62%)	47(64.38%)	
College or undergraduate education	525(58.01%)	404(76.95%)	121(23.05%)		107(43.67%)	34(31.78%)	73(68.22%)	
Postgraduate or above	32(3.54%)	26(81.25%)	6(18.75%)		5(2.04%)	2(40.00%)	3(60.00%)	
Monthly household income per capita (CNY¥)				0.009				0.761
< 5000	126(13.92%)	83(65.87%)	43(34.13%)		44(17.96%)	18(40.91%)	26(59.09%)	
5000–7999	233(25.75%)	144(61.80%)	89(38.20%)		68(27.76%)	20(29.41%)	48(70.59%)	
8000–9999	137(15.14%)	99(72.26%)	38(27.74%)		45(18.37%)	16(35.56%)	29(64.44%)	
10,000–14,999	163(18.01%)	122(74.85%)	41(25.15%)		34(13.88%)	12(35.29%)	22(64.71%)	
15,000–19,999	99(10.94%)	67(67.68%)	32(32.32%)		27(11.02%)	7(25.93%)	20(74.07%)	
> 20,000	147(16.24%)	115(78.23%)	32(21.77%)		27(11.02%)	8(29.63%)	19(70.37%)	
Allergy in children				< 0.001				0.025
Yes	136(15.03%)	126(92.65%)	10 (7.35%)		22(8.98%)	12(54.55%)	10(45.45%)	
No	769(84.97%)	504 (65.54%)	265(34.46%)		223(91.02%)	69(30.94%)	154(69.06%)	
Congenital diseases in children				0.979				0.154
Yes	10(1.10%)	7(70.00%)	3 (30.00%)		1(0.41%)	1(100.00%)	0(0.00%)	
No	895(98.90%)	623 (69.61%)	272(30.39%)		244(99.59%)	80(32.79%)	164(67.21%)	

Similarly, as shown in Table 1, there were 245 children who had received only the first dose of the COVID-19 vaccine. Among them, over half of the parents (62.86%) were in the 30–40 age group. The majority of parents had a college or undergraduate degree (43.67%), and 46.13% of families had a monthly per capita income between 5000 and 9999. The majority of preschool children (91.02%) did not have any allergies, and 99.59% did not have any congenital diseases.

### Correlation analysis of sociodemographic variables and parental hesitancy towards COVID-19 vaccination for preschool children

As shown in Table 1, among the 905 parents who had never vaccinated their children against COVID-19, 275 parents (30.4%) reported no vaccine hesitancy for the first dose of the COVID-19 vaccine for preschool children, while 630 parents (69.6%) reported hesitancy. Single-factor correlation analysis of all sociodemographic variables found that parental vaccine hesitancy for the first dose of COVID-19 vaccine for preschool children was significantly associated with parental age (*p* = 0.006), kindergarten year of children (*p* = 0.043), parental education level (*p* < 0.001), monthly household income per capita(*p* = 0.009), and presence of allergies in children (*p* < 0.001).

Similarly, as shown in Table 1, among the 245 parents who had vaccinated their preschool children with only one dose of the COVID-19 vaccine, 164 parents (66.9%) reported no vaccine hesitancy for the second dose of COVID-19 vaccine, while 81 parents (33.1%) reported hesitancy. Single-factor correlation analysis of all sociodemographic variables found that parental vaccine hesitancy for the second dose of COVID-19 vaccine for preschool children was significantly associated with parental age (*p* < 0.001) and presence of allergies in children (*p* = 0.025).

### Reasons for parental hesitancy towards COVID-19 vaccination for preschool children

As shown in Table 2, among the reasons for parental vaccine hesitancy reported by parents for the first and second doses of COVID-19 vaccine for preschool children, 79.2% and 85.2% of parents, respectively, chose "I am worried about the side effects of the vaccine," and vaccine safety became the primary concern of parents. In the hesitancy for the first dose of COVID-19 vaccine, nearly half of the parents (44.8%) selected "there are many negative news reports about the vaccine." For the hesitancy regarding the second dose of COVID-19 vaccine, more than half of the parents (53.1%) chose this reason. Among parents hesitating for the first dose and the second dose of the vaccine, 37.3% and 27.2% of parents, respectively, selected "my child is not in good health and cannot receive the vaccine," while 34.1% and 38.3% of parents chose "I am concerned that this vaccine may not effectively protect my child."

**Table 2:** Reasons why parents hesitate to vaccinate preschool children against COVID-19.

Reasons for vaccine hesitancy	First does (n = 630) (%)	Second does (n = 81) (%)
I am worried about the side effects of the vaccine	499(79.2)	69(85.2)
There has been a lot of negative news about vaccines	282(44.8)	43(53.1)
The child is not well enough to be vaccinated	235(37.3)	22(27.2)
I am worried that the vaccine is not effective in protecting children	215(34.1)	31(38.3)
The child is allergic to the vaccine	97(15.4)	13(16.0)
The child is healthy enough not to be vaccinated	72(11.4)	7(8.5)
Others	98(15.6)	11(13.6)

Percentages sum to > 100% because respondents were asked to check all reasons that applied to their decision.

### Logistic regression analysis of factors associated with parental hesitancy for COVID-19 vaccination in preschool children

As demonstrated in Tables 3 and 4, logistic regression analyses were employed to explore the relationships between sociodemographic variables, perceived social support variables, health literacy variables, and parental hesitancy for the first and second doses of COVID-19 vaccination in preschool children.

**Table 3:** Logistic correlation analysis of the relationship between first-dose COVID-19 vaccine hesitancy and socio-demographic factors, perceived social support and health literacy variables in preschool children.

Variables	First does of COVID-19 Vaccine (N = 905)								
	Model 1			Model 2			Model 3		
	OR	95% CI	*P*	OR	95% CI	*P*	OR	95% CI	*P*
Parental age	1.20	0.91,1.58	0.204	1.13	0.85,1.53	0.390	1.11	0.82,1.49	0.514
Kindergarten year	1.00	0.83,1.21	0.990	1.06	0.87,1.29	0.580	1.05	0.86,1.29	0.640
Parental educational level	1.47	1.20,1.79	< 0.001	1.59	1.28,1.96	< 0.001	1.64	1.31,2.04	< 0.001
Monthly household income per capita (CNY¥)	1.04	0.94,1.14	0.487	1.09	0.98,1.20	0.122	1.10	0.99,1.22	0.080
Allergy in children	0.18	0.09,0.36	< 0.001	0.15	0.07,0.30	< 0.001	0.14	0.07,0.29	< 0.001
Information acquisition				1.07	0.75,1.52	0.723	1.13	0.79,1.63	0.509
Information comprehension				1.40	1.01,1.94	0.044	1.40	1.01,1.95	0.045
Information communication				0.87	0.72,1.05	0.146	0.88	0.73,1.06	0.173
Information decision-making				0.27	0.18,0.41	< 0.001	0.27	0.18,0.41	< 0.001
PSSO							0.90	0.67,1.22	0.501
PSS-Fa							1.35	1.00,1.83	0.047
PSS-Fr							0.72	0.54,0.95	0.022
R^2^	0.066			0.149			0.156		
F	73.48			165.32			173.75

**Table 4:** Logistic correlation analysis of the relationship between second-dose COVID-19 vaccine hesitancy and socio-demographic factors, perceived social support and health literacy variables in preschool children.

Variables	Second does of COVID-19 Vaccine (N = 245)								
	Model 1			Model 2			Model 3		
	OR	95% CI	*P*	OR	95% CI	*P*	OR	95% CI	*P*
Parental age	2.74	1.60,4.70	< 0.001	2.82	1.59,5.00	< 0.001	2.87	1.61,5.10	< 0.001
Allergy in children	0.40	0.16,0.99	0.049	0.37	0.13,1.05	0.061	0.39	0.14,1.12	0.080
Information acquisition				0.56	0.27,1.16	0.119	0.54	0.26,1.13	0.102
Information comprehension				1.63	0.86,3.08	0.131	1.63	0.86,3.09	0.136
Information communication				0.90	0.64,1.28	0.569	0.87	0.61,1.24	0.444
Information decision-making				0.41	0.19,0.86	0.019	0.39	0.18,0.84	0.016
PSSO							0.99	0.57,1.72	0.967
PSS-Fa							1.16	0.65,2.08	0.607
PSS-Fr							1.11	0.64,1.92	0.712
R^2^	0.063			0.153			0.159		
F	19.55			47.50			49.44		

Analyzing the factors contributing to parental hesitancy for the first dose of COVID-19 vaccine in preschool children, Model 1 controlled for parental age, child's kindergarten grade, parental education level, average monthly household income, and whether the child had any allergies, explaining 6.6% of the variance (F = 73.48, *p* < 0.001). When Model 2 incorporated HL variables, the proportion of explained variance increased to 14.9% (F = 165.32, *p* < 0.001). In Model 3, PSS variables were included and accounted for 15.6% of the variance (F = 173.75, *p* < 0.001). The results revealed significant associations between parental education level (OR = 1.64, *p* < 0.001), presence of allergies in children (OR = 0.14, *p* < 0.001), information comprehension (OR = 1.40, *p* = 0.045), information decision-making (OR = 0.27, *p* < 0.001), PSS-Fa (OR = 1.35, *p* = 0.047) and PSS-Fr(OR = 0.72, *p* = 0.022)with parental hesitancy for the first dose of COVID-19 vaccine in preschool children.

Analyzing the factors contributing to parental hesitancy for the second dose of COVID-19 vaccine in preschool children, Model 1 controlled for parental age and whether the child had any allergies, explaining 6.3% of the variance (F = 19.55, *p* < 0.001). When Model 2 incorporated HL variables, the proportion of explained variance increased to 15.3% (F = 447.50, *p* < 0.001). In Model 3, PSS variables were included and accounted for 15.9% of the variance (F = 49.44, *p* < 0.001). The results showed a significant association between parental age (OR = 2.87, *p* < 0.001) and information decision-making (OR = 0.39, *p* = 0.016) with parental hesitancy for the second dose of COVID-19 vaccine in preschool children.

## Discussion

The results of this study show that regardless of the first or second dose of the COVID-19 vaccine, "concerns about vaccine side effects" were the primary factor leading to parental hesitancy in vaccinating preschool children against COVID-19. The models indicated that parental education level, presence of allergies in children, information comprehension, information decision-making, family social support (PSS-Fa) and friends social support (PSS-Fr) were associated with parental vaccine hesitancy for the first dose of the COVID-19 vaccine. For the second dose, parental age and information decision-making were associated with parental vaccine hesitancy.

Previous research has reported vaccine hesitancy rates among parents of 55% in Italy^23^, 25% in Ireland^26^, 26% in the UK^26^, 33% in the US^26^, and 55% in Turkey^25^, A study conducted in Taizhou, China, revealed a vaccine hesitancy rate of 45.2% among parents^44^. In our study, the hesitancy rate for the first dose of the COVID-19 vaccine in preschool children was 69.6%, and for the second dose, it was 33.1%. The hesitancy rate for parents to administer the first dose of the COVID-19 vaccine to preschool children is higher than that for the second dose. This may be attributed to parents' lack of accurate awareness regarding COVID-19 vaccine-related information, resulting in a lack of trust in the COVID-19 vaccine. Conversely, parents who have already vaccinated their children with the first dose have established preliminary trust in the vaccine. Therefore, intervention programs targeting parents of preschool children for COVID-19 vaccine administration should prioritize those who have never administered the COVID-19 vaccine to their children, aiming to alleviate parental concerns. However, it is also essential to provide guidance to parents who have not completed the full vaccination course, offering recommendations for the future implementation of policies regarding comprehensive COVID-19 vaccine booster shots. With the implementation of policies for administering the full dose of the COVID-19 vaccine to children, it is crucial to address vaccine hesitancy, particularly for the first dose. Furthermore, focusing on the factors influencing vaccine hesitancy for different doses can help in implementing targeted intervention measures.

The results of this study demonstrate that in the univariate analysis of sociodemographic factors, parental age, kindergarten year of children, parental education level, and monthly household income per capita are associated with parental vaccine hesitancy regarding the COVID-19 vaccine. These findings are consistent with previous research^25,44^. Specifically, for the administration of the first dose of the COVID-19 vaccine to children, a higher parental age is correlated with a higher vaccine hesitancy rate. This association may be attributed to lower acceptance of vaccine information among older parents^25^. It is noteworthy that the kindergarten year of children is associated with the administration of the first dose of the COVID-19 vaccine but not with the second dose. A higher grade typically implies older age for children, and most children who have not received the first dose are usually in the primary class. The Chinese health authorities have opened COVID-19 vaccination for children and adolescents aged 3–17. Therefore, parents might consider their child being too young for vaccination. Importantly, children with allergic diseases evoke parental hesitancy, regardless of whether it is for the first or second dose of the COVID-19 vaccine, aligning with previous research findings^28^.

The reasons for parental vaccine hesitancy, as reported by themselves in this study, indicate that the primary reason for vaccine hesitancy, whether it is for the first or second dose of the COVID-19 vaccine, is concerns about vaccine safety and potential side effects, which is consistent with previous research^28^. Additionally, the abundance of negative news related to vaccines on social media significantly impacts parental confidence in vaccinating their children against COVID-19 and serves as the second major reason reported in this study. Previous studies have also demonstrated that parental mistrust of vaccines may stem from a lack of correct understanding regarding vaccine safety and negative media coverage of vaccines^45^. All these reasons are associated with parents' lack of accurate knowledge about the COVID-19 vaccine. Parents' main concerns are based on insufficient understanding of how vaccines work, the adoption of misinformation about vaccines, and ignorance of the severe health consequences that children may face if infected with COVID-19^46^. Therefore, healthcare professionals play a crucial role in educating and providing accurate information regarding the benefits and risks of vaccines. Offering comprehensive information about the safety and efficacy of COVID-19 vaccines may be a key strategy to reduce vaccine hesitancy, increase vaccine demand, and improve actual vaccination rates^28,45,47,48^. Public health strategies should call upon hospitals, communities, and healthcare professionals to conduct targeted awareness campaigns through various media channels. It is essential to provide clear information about the safety of vaccines and disseminate accurate information regarding vaccine side effects. In this study, the dimensions of health literacy, specifically information comprehension and information decision-making, were found to be associated with parental hesitancy towards vaccinating their children with the first dose of the COVID-19 vaccine. Information decision-making was also correlated with parental hesitancy towards the second dose of the vaccine. The measurement of health literacy explored parents' personal abilities to acquire and understand health information related to vaccines. Parental health literacy can be reflected in the process of obtaining COVID-19 vaccine-related information through various channels such as social media and interpersonal communication. Subsequently, parents assess the susceptibility of children to COVID-19 and the protective effects of the vaccine. They then make decisions regarding the vaccination of their children through communication and interaction with peers and healthcare professionals^36,38^. Information comprehension represents parents' understanding of the susceptibility and severity of COVID-19, as well as their awareness of the safety and effectiveness of the vaccine. Consistent with previous research, attitudes towards vaccines are associated with vaccine hesitancy, as parents who hold positive views towards vaccines are more willing to have their children vaccinated^25^. Information decision-making refers to the process in which parents make decisions about vaccinating their children based on the vaccine-related information they have obtained and external influences. Given the greater uncertainty in health communication^31,49^, the ability to distinguish reliable and unreliable information is crucial in shaping health outcomes, such as health behaviors. Selective trust in information sources (particularly trusting formal sources while distrusting informal sources) has been found to be associated with vaccine willingness^31^. This suggests the need to utilize formal sources such as health departments and healthcare professionals as channels for disseminating COVID-19 vaccine information, thus avoiding the spread of false and inaccurate information to parents^31^. Health departments should issue official guidelines to ensure that parents can access accurate information promptly. The government should leverage community health service centers to organize health education events for parents, facilitating dialogues between parents and healthcare professionals to enhance their capacity for collecting and discerning health information. Parents should also exert self-efficacy and enhance their own health literacy throughout this process, thereby reducing hesitancy towards vaccinating their children against COVID-19.

According to the survey results of this study, perceived social support from family and friends is associated with parental hesitancy towards administering the first dose of the COVID-19 vaccine to children, consistent with findings from previous research. Family and friends play a relevant role in shaping individuals' attitudes towards COVID-19 vaccines, highlighting the impact of perceived social support. The measurement of perceived social support explores the ability of parents to obtain external support to enhance their self-efficacy in making health decisions regarding COVID-19 vaccine administration for their children. Throughout the process of vaccinating children against COVID-19, parents can acquire information about the benefits of the vaccine in preventing COVID-19 infections in children and the vaccination process through channels such as friends and family. The support and encouragement received through conversations can strengthen their confidence in vaccinating their children against COVID-19, thus reducing vaccine hesitancy. This may be attributed to the sense of connectedness with social networks, which facilitates information gathering, increases self-efficacy, and encourages engagement in preventive actions^24^. The emergence of the COVID-19 infection as a sudden public health event instills vigilance among parents. Social support serves not only as a buffer against distress but also enables recipients to optimize their adaptability by enhancing self-efficacy^34^. Therefore, when it comes to the decision of whether to vaccinate their own children against COVID-19, external information support is necessary. Seeking shared experiences becomes a significant requirement for parents. Moreover, information trusted by parents often comes from family and friends. Research findings suggest that a more effective approach to encouraging vaccination is through alternative channels that are trusted by these individuals, particularly informal sources such as friends and family^31,49^. This finding supports the existing literature on the importance of informal social networks in influencing health behaviors^50^. Therefore, in vaccination efforts, community healthcare workers, family members, and friends should actively share the benefits and experiences related to receiving the COVID-19 vaccine. Communities should provide convenient vaccination pathways, express support and encouragement for parents to vaccinate their children against COVID-19, and enhance parental confidence. Trusted figures in the medical field should use social media to encourage parents to vaccinate their children, thereby improving the effectiveness of vaccination campaigns. Perceived social support does not significantly influence hesitancy towards the second vaccine dose, which may be attributed to parents already having received the COVID-19 vaccine themselves.

^40,422834,533434^This study has several limitations. Firstly, convenience sampling was employed, and participants were recruited from the Minhang District in Shanghai, which is a region with a relatively high level of economic development and urbanization. The similarity in participants' living environments and economic levels may limit the generalizability of the research findings to other regions, particularly economically underdeveloped rural areas. Future studies should adopt more rigorous sampling methods to obtain a nationally representative sample and a more balanced sociodemographic population to determine vaccine hesitancy and its influencing factors. Secondly, parents who have administered two doses of the COVID-19 vaccine to their children may still experience vaccine hesitancy, a category not included in this study. In future studies, we will comprehensively consider the influencing factors for different populations to obtain more scientifically grounded conclusions. Third, the results of this study are based on self-reported information, which may introduce information bias. The data for this study were collected through online surveys, which means there may be some circumstances that affect the quality of the data, such as interpretation biases of questionnaire content or participants filling out the questionnaire without reading it. To address this, we implemented logical test questions to prevent participants from filling out the questionnaire without reading it. Lastly, this study employed a cross-sectional observational design, thus causal conclusions cannot be drawn.

## Conclusion

This cross-sectional study was conducted in Shanghai, China, to measure the influence of perceived social support and health literacy on parental COVID-19 vaccine hesitancy among parents of preschool children. The study revealed a high level of vaccine hesitancy among parents of preschool children regarding the COVID-19 vaccine. A lack of accurate and scientifically informed understanding of vaccine information among parents was identified as a significant factor contributing to vaccine hesitancy. The findings of this study provide evidence regarding the factors influencing parental COVID-19 vaccine hesitancy and offer guidance for better managing vaccine hesitancy phenomena, formulating COVID-19 vaccine immunization strategies, and establishing effective health education models to increase parents' confidence in vaccinating their children.

## Data Availability

The datasets used to support the findings of this study are available from the corresponding author on reasonable request.

## Abbreviations

VH: Vaccine hesitancy
PSS: Perceived social support
HL: Health literacy
